# MicroRNA-138 inhibits migration and invasion of non-small cell lung cancer cells by targeting LIMK1

**DOI:** 10.3892/mmr.2021.12209

**Published:** 2021-06-08

**Authors:** Yanjuan Tan, Huaidong Hu, Wuyuan Tan, Longyu Jin, Jianxin Liu, Hui Zhou

Mol Med Rep 14: 4422-4428, 2016; DOI: 10.3892/mmr.2016.5769

Following the publication of this paper, it was drawn to the Editors’ attention by a concerned reader that certain of the western blotting data shown in Figs. 3C and 5A were strikingly similar to data appearing in different form in another article by different authors, which had already been published elsewhere at the time of the present article's submission. Furthermore, cell Transwell assay data in the article (featured in Fig. 4B) were strikingly similar to data appearing in different form in other articles by different authors, which were either already under consideration for publication or had already been published elsewhere at the time of the present article's.

Owing to the fact that the contentious data in the above article were either already under consideration for publication, or had already been published elsewhere, prior to its submission to Molecular Medicine Reports, the Editor has decided that this paper should be retracted from the Journal. The authors were asked for an explanation to account for these concerns, but the Editorial Office never received any reply. The Editor apologizes to the readership for any inconvenience caused.

